# The factors affecting male infertility: A systematic review

**DOI:** 10.18502/ijrm.v19i8.9615

**Published:** 2021-09-09

**Authors:** Hamideh Jafari, Khadigeh Mirzaiinajmabadi, Robab Latifnejad Roudsari, Marzieh Rakhshkhorshid

**Affiliations:** ^1^Student Research Committee, Mashhad University of Medical Sciences, Mashhad, Iran.; ^2^Nursing and Midwifery Care Research Centre, Mashhad University of Medical Sciences, Mashhad, Iran.; ^3^Department of Midwifery, School of Nursing and Midwifery, Mashhad University of Medical Sciences, Mashhad, Iran.; ^4^Department of Midwifery, Nursing and Midwifery School, Zahedan University of Medical Sciences, Zahedan, Iran.

**Keywords:** Male, Infertility, Sterility, Urogenital diseases.

## Abstract

**Background:**

In recent years, the incidence of male infertility has increased worldwide. It is necessary to study the factors that influence male infertility in each area/region for better management.

**Objective:**

To determine the factors affecting male infertility in the Iranian male population.

**Materials and Methods:**

An online search was conducted in electronic databases including PubMed, Google Scholar, SID, and Scopus to identify articles on the factors associated with male infertility, published in English and Persian. The keywords used to perform the search included “factor", “epidemiology", “causes of infertility", and “male infertility". The search was conducted without a time restriction, up to April 2020.

**Results:**

The search resulted in a total number of 691 studies. After an assessment of the articles, finally 14 studies were included in this study with a total number of 26,324 infertile males. The factors associated with male infertility included semen abnormalities, varicocele and testis disorder, smoking, exposure to heat, obesity, anabolic steroids, vascular abnormalities, anti-spermatogenesis factors, antidepressants, taking ranitidine and cimetidine, penile discharge and genital ulcers, painful micturition, occupational factors, alcohol, chronic disease, sexual disorder, Surgical and urological diseases, genetic factors and herpes infection. Among these, the semen and varicocele disorders were common in most studies.

**Conclusion:**

The present review suggests that the factors affecting male infertility in Iran are similar to those reported from other countries. The results of this study can be used in adopting appropriate strategies for infertility management in Iran.

## 1. Introduction

Infertility and its treatment are important global concerns that have different social dimensions (1-4). Infertility is the inability of a couple to get pregnant within one year of regular unprotected intercourse. The prevalence of primary infertility among couples is 13.2-17.3% (5, 6). The main causes of primary infertility are the ovulatory problem (39.7%) and male factors (29.1%) (6). At least half of all infertility cases are related to male infertility (7). Jafari estimated the prevalence of male factors as 40.9% (8). In another study, the distribution of infertility due to male factors was reported to range from 20-70% and the percentage of infertile men from 2.5 to 12% (9). Evidence indicates that male infertility has increased in some populations (10). Worldwide studies have shown that the increasing infertility in recent years could be due to anatomical, physiological, and genetic factors. Many environmental conditions and acquired factors, such as smoking and alcohol consumption, changes in sexual behavior, and diet, also influence fertility and semen quality, and therefor can lead to the diversity in etiology and patterns of infertility in different regions (6, 11-13). Male infertility is a global health concern and its management is important because it not only affects the infertile couple but also the phenomenon of childbearing as a whole. Understanding the factors related to male infertility in each region separately can help healthcare providers and policymakers to plan appropriate strategies for its management. Without accurate and precise data from the region, planning to comprehensively identify and treat infertile men is not possible (14). Therefore, this systematic review aimed to determine the factors affecting male infertility in Iran.

## 2. Materials and Methods

In this systematic review, articles were identified in the following databases: PubMed, Google Scholar, Scopus, SID, and Magiran. The search was performed using the following keywords: “factor,” “epidemiology,” “causes of infertility,” “male infertility,” “fertility,” and “infertility” in English and Persian. The search was conducted without a time restriction, up to April 2020.

All relevant studies and references were searched to detect additional articles, and the quality of the articles was evaluated using the Joanna Briggs Institute Critical Appraisal tools (15). All collected articles were reviewed and some were excluded based on the title, abstract, or duplication. Then, selected studies were assessed based on the inclusion and exclusion criteria of the study.

The Inclusion criteria were research on infertile male participants, conducted in Iran, and reporting human studies. The exclusion criteria were infertile women with fertile/normal male participants, duplicate paper, review studies, animal studies, infertile couples with unspecified gender, and abstracts presented in congresses.

Data were extracted independently by two reviewers. Disagreement between the researchers were resolved through discussion. The following information was extracted from each study: the authors' names, date of publication, study design, reported results, and total number of infertile men.

## 3. Results

**Figure 1:**
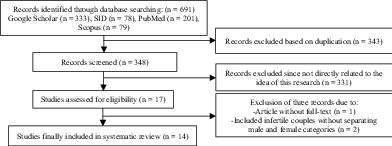
Outline of the article search process.

A total of 691 articles were found through the database search. After an assessment of the titles and abstracts, 674 duplicates and irrelevant studies were excluded and 17 articles were assessed for eligibility. At this stage, 3 articles were excluded from the study process, too, one article due to inaccessibility of the full-text and two articles due to the inclusion of infertile couples without separating male and female categories. Finally, 14 studies were included in this study including retrospective, cohort, and cross-sectional studies (Table I, Figure 1). The quality assessment of the articles was done using the Joanna Briggs Institute Critical Appraisal checklist.

A total of 26,324 infertile men were studied in these articles, which were published between 2007 and 2018.

While the study of Ahmadi with 96 participants had the lowest sample size, the study of Safari Nejad with 12,284 infertile men had the largest (16, 17). The factors related to male infertility included semen abnormalities (12, 18-24); genetic factors (12); vascular abnormalities (12); anti-spermatogenesis factors (12, 22, 25); cryptorchidism; varicocele, and testis disorder (16-23, 25-27); smoking and lead exposure (17, 22, 23, 25, 27); exposure to heat (17); obesity (17, 22, 23); anabolic steroids (17, 22); antidepressants; penile discharge, painful micturition, and genital ulcers (17); occupational factors (17, 21); alcohol consumption (25); chorionic disease (16, 20); sexuality disorder (17, 19); surgery record (16, 20, 25); ranitidine (23); cimetidine (25); urinary tract infection (27); and herpes infection (28). Among of the mentioned factors, semen and varicocele disorders were common in most studies. The studies have been summarized in Table I.

**Table 1:** General features of the studies


**Authors, year (Ref number)**	**Center**	**Sample size**	**Object**	**Result${}^{\#}$**
**Karimpour, 2006 (26)**	Infertility centers in Mazandaran	2,235	Determine the incidence of varicocele in men with infertility	Varicocele (42.6)- Congenital bilateral absence of the vas deferens (1.8)- Testicular torsion (0.1)- Cryptorchidism (14)
**Kamali, 2007 (18)**	Royan Institute in Tehran	2,492	Survey the epidemiology of infertility in Royan Institute	Semen abnormalities (63.9)- Azoospermia (23.6)- Varicocele (21)
**Safarinejad, 2008 (17)**	A population-based study in Iran	12,285	Prevalence and risk factors of infertility in Iran	Varicocele (16)- Cryptorchidism (1.5)-Current smoker (42)- Ex-smoker (16)- BMI-Male exposure to heat (28)- Anabolic steroids (3.5)- Antidepressants (22)- penile discharge, painful-micturition, and genital ulcers (8.8)
**Karimpour Malekshah, 2011 (19)**	Infertility Clinics in Mazandaran	3,734	Determine the clinical patterns and major causes of infertility in Mazandaran	Semen factor (50.5)- Varicoceles (42.7)- Deferens agenesis (1.2)- Cryptorchidism (0.5)- Testicular torsion (0.1)- Miscellaneous male factors (hypospadias, retrograde ejaculation, coital problem) (3.1)
**Taghavi, 2011 (20)**	Urology Clinic of Imam Reza Hospital of Mashhad	2,000	Determine the clinical major causes of infertility in Mashhad	Varicocele (37.4)- Testis atrophy (6.15)- Ectopic testis (1.25)- Spermatocele (0.95)- Gynecoplasty (0.3)- Hydrocele (0.65)- Inguinal hernia (0.35)- Sperm abnormality (55.2)- Azoospermia (21.7)- Chronic disease (3.65)
Choobineh, 2013 (15)	Shariati Hospital Infertility Clinic, Tehran	200	Examine the demographic characteristics of infertile men	Dealt with chemicals in worker (26)- Varicocele decreased sperm motility (81)- Decrease in sperm count (42)- Azoospermia (46)
**Keshavarzi, 2013 (22)**	Infertility Center of Mo'tazedi Hospital in Kermanshah	514	Determine the associated risk factors of infertility	Varicocele (1.15)- Azoospermia (3.89) BMI-smoking (55.3) - Anabolic steroids (3.5)
**Monavari, 2013 (28)**	Clinical Center for Infertility in Yazd	70	Determine the prevalence of HSV-1 and HSV-2 using real-time PCR in the semen of a randomized asymptomatic infertile male	HSV infection in semen HSV-1 (22.9) HSV-2 (14.3)
**Ahmadi, 2014 (16)**	Infertility clinics of Ilam province	96	Determine associated factors with male infertility by using semen analysis	Surgical and urological diseases (45.8)
**Mahboubi, 2014 (23)**	Infertility clinic in Shiraz	268	Determine the most common risk factors for male infertility in Iranian men	Varicocele (36)- Azoospermia (30.6)- Smoking (32.4)- Taking ranitidine (14.8)- Cigarette smoking (32.4)– BMI*- Job (62)- Teratospermia (84)- Asthenospermia (82.7)- Oligospermia (52.7)- Hernia (36)
**Sohrabvand 2015 (25)**	Infertility clinics of Vali-e-Asr, Tehran	430	Clarify the associated factors that might play a role in Iranian infertile men	Cigarette smoking (29)- Varicocele operation (22)- Hernia operation (6.2)- Orchiopexy operation (4.2)- Undescended testis (6.2)- Opium addiction (9.2)- Alcohol consumption (5.5)- Cimetidine (0.9)- Lead exposure (0.6)
**Masoumi, 2015 (13)**	Infertility center in Fatemieh Hospital in Hamadan	1,200	Determine the frequency of the causes of infertility in infertile couples	Semen abnormalities (44.6)- Genetic factors (29.8)- Anti-spermatogenesis agents (11)- Vascular disorders (17.2)
**Shafabakhsh, 2017 (27)**	Clinical Center for Infertility in Yazd	600	Determine the influence of infertility, smoking, alcohol, and addictive drugs	Varicocele (20.7)- Smoking (34)- Urinary tract infection (6.3)- Hookah (5.7)- Opium (19)- Naswar (0.7)
**Hashemzadeh, 2018 (24)**	Infertility clinics of Vali-e-Asr, Tehran	200	Determine the relationship between body mass index and quality of sperm parameters	Oligospermia (34)- Asthenospermia (27.5)- Teratospermia (3)
${}^{\#}$Numbers are presented as percentages, *The difference between average BMI was statistically significant in both fertile and infertile groups p < 0.05. BMI: Body mass index, HSV: Herpes simplex virus				

## 4. Discussion

This systematic review investigated the causes of male infertility. Semen parameters and varicocele were included in most studies (12, 16, 18-27). The prevalence of varicocele in men with infertility problems is twice that of the normal population. In Karimpur's study in 2011, varicocele was responsible for 42.7% of cases of infertility in males (19). The higher prevalence of varicocele in the infertile men population is the most important evidence supporting the theory of the relationship between varicocele and male infertility (29). Varicocele can lead to an increase in the temperature of the testes and cause reflux of toxic metabolites from the adrenal vein to the left kidney (30).

Li and Skakkebaek indicated that “according to many studies the semen quality in men is declining throughout the world and during the past decades a possible degradation in human semen quality has become an important public health issue" (31, 32). In a nine-year study, one in five investigated men (1737/8518) was diagnosed with reduced sperm counts (33). In 50% of infertile couples, semen disorders is the male infertility-associated influence (34). A study conducted in China reported that men's lifestyles there are related to their sperm quality (35). Durairajanayagam found that “a wide variety of risk factors could potentially influence sperm quality. These include lifestyle factors such as cigarette smoking, alcohol intake, use of illicit drugs, anabolic–androgenic steroids, obesity, psychological stress, diet, and caffeine intake" (36). In the present evaluation of studies, smoking and obesity were among the factors affecting infertility. Perhaps one of the reasons for this is their impact on the quality of sperm, which has been mentioned in several studies. Kazemijaliseh found that aging, higher body mass index (BMI), active smoking, and higher educational levels are associated with increased infertility (6). Obesity is associated with lower serum testosterone and luteinizing hormone, elevated rates of oligospermia or azoospermia, decreased semen volume, and sperm concentration. It seems that multiple factors are involved in the relationship between obesity and male infertility (37), including that obesity can affect male fertility through erectile dysfunction. Many studies have demonstrated the adverse effects of smoking, alcohol drinking, and weight on sperm; only a few observed no effect on any semen parameters (31, 38-40). For example, Hashemzadeh indicated that there was no significant relationship between the BMI in three groups of men (grouped based on BMI and their overall sperm analysis (counts, motility, and shape) (24). Dechanet reported that “obesity and cigarette smoking were factors associated with decreased fertility by causing a delay in conception and decreased in vitro fertilisation results" (41).

According to the studies, tobacco smoking has many negative effects on semen parameters (i.e impaired motility of spermatozoa, lower semen concentration, and increased morphology defects in tail, neck, and head) resulting in male infertility (10, 42-46).

The cigarette toxic contents might have adverse effects on the developmental processes of the male germ cells (10, 47, 48). Also, products in alcohol and cigarettes that cause oxidative stress could be have a role in the pathogenesis of birth defects, embryo development, moles, cancers, preeclampsia and preterm labor, thus might lead to abortion and infertility (49).

Other factors that can affect male infertility are environmental and occupational agents that may result in genetic disorders in gonadal cells (50, 51).

The association of toxic materials as an environmental factor with infertility has been considered (52). In our review, some studies indicated the influence of occupational and environmental factors on infertility (17, 21, 22). Sedentary jobs are frequently associated with testicular overheat and possible risk of sperm DNA harm (3, 53-55).

Other factors associated with male infertility in our study were herpes infection (28) and urinary tract infection (27). According to these studies, asymptomatic seminal infection with herpes simplex virus adversely affects the sperm count (28). Punab's study indicated that “the majority of azoospermia cases had epididymis obstruction, which has most probably been caused by sexually transmitted diseases in the past" (33). Infectious diseases such as inflammatory reactions within the male genital tract are responsible for a greater proportion of infertility (7, 56).

Based on the available scientific evidence, it is not possible to say with certainty whether these are true “potential contributing factors” or a result of impaired physiology and health. More high-quality research with well-designed studies is needed to reach a strong evidence base on the factors affecting male fertility and for developing treatment guidelines in this context (57-59). However, in most of the studies, semen and varicocele disorders were common problems that therefore warrant further consideration. Also, smoking and obesity are factors that can be reduced through behavioral change. It is necessary to assess lifestyle patterns of infertile men before and during infertility treatments and to implement healthy lifestyle counseling programs for them.

## 5. Conclusion

The present review suggests that the factors affecting male infertility in Iran are similar to those reported in other countries. The results of this study can be used in planning appropriate strategies for the management of infertility in Iran.

##  Conflict of Interest 

None declared.
